# Baltimore Academy of Medicine—a Case of Uncontrollable Epistaxis

**Published:** 1888-08

**Authors:** 


### BALTIMORE ACADEMY OF MEDICINE.

### Regular Meeting Held Dec. 6, 1887. A Case of Uncontrollable Epistaxis.

Dr. J. J. Chisolm reported the case of a lad sixteen years old who from his first year had had nose-bleeding His mother said the nurse was throwing him about and struck his nose, and that from that time the hemorrhages had been abundant and fearful. When brought to Dr. Chisolm, the boy had a hyperæmic state of the nasal mucous membrane and some discharge, and on using a douche to remove the discharge a hemorrhage was started which nearly exsanguined the boy. The mother said this was the usual condition; that he lost color, became pallid, dull and stupid, but recuperated quick y, and by the time he recovered another attack would come on and the blood would flow in an actual stream for three or four hours at a time, the left nostril bleeding more than the right. As he was the son of a physician (now dead) nothing had been left undone to help him. Ergot and iron would stop the hemorrhage, but so would time.

Nothing was of use. If he did not bleed for three or four weeks, he became lethargic and sleepy, and after losing about twenty ounces of blood he felt bright and well the next day. The bleeding happened on an average of twice a month, somtimes occuring again after a lapse of three or four days and sometimes with the intervals of a month. He very often bled to fainting. Dr. Chisolm had tried every known remedy to prevent the attacks, but without avail, and even went so far as to use the old-fashioned treatment of putting a blister over the hepatic region.

DISCUSSION.

Dr. G. Lane Taneyhill said he had had three cases resembling in some respects that of Dr. Chisolm's. The trouble was not so much to stop the bleeding as to prevent it. and treat the patient in the interim. He had tried many things and found that 5 drops of the oil of turpentine in milk three times a day, together with keeping the head cool, the bowels open, not allowing too active exercise and avoiding overloading the stomach had accomplished good recults. These three cases were now in the eighth month of treatment and were doing well. He had previously used ergotin with little benefit.

Dr. F. T. Miles asked if the galvano-cautery had been used. He thought that a thorough cicatrization of the mucous membrane was a good and not too severe treatment. and in this way the large venous sinuses could be easily destroyed. The turbinated bones were also now often extirpated as a remedy.

Dr. J. J. Chisolm replied that he had been removing turbinated bones for many years back, and did not consider it an operation to fear. This was not the ordinary nasal hemorrhage of youth, and it was no hereditary diathesis. He had had another case not long since of hemorrhagic diathesis in which a slight scratch on the finger with a pin had caused bleeding for an hour. In the first case there was no special rhinoscopic appearance.

Dr. F. T. Miles remarked that no constituent (except the hair and nails) of the body was reformed so rapidly as the blood, and again under some circumstances it seem d impossible for the blood to be renewed.

Dr. S. C. Chew was recently called to see a young lady whom he had known all her life. She had had an upper molar tooth extracted that morning and when he arrived the blood was flowing in a stream. Alum and cotton soaked with the persulphate of iron had been used and it was still oozing. She had probably lost as much as a half gallon of blood. He took off everything and applied cotton compresses soaked with the persulphate of iron, when the bleeding stopped. She was extremely blanched, but the next day he saw her and she was as well as ever.

He then reported a case of

HYDROPHOBIC TETANUS.

The case occurred in the practice of Dr. William Green and was exceedingly rare. When he saw it there was great difficulty in swallowing and an averseness to attempt to swallow. The reflex irritation of the vessel to the lips brought on spasmodic action. The nozzel of a tea-pot was wrapped with cotton and introduced behind the molar tooth and nourishment was given. The treatment has been chloral, bromide and morphia hypodermically. The etiology was obscure. There was no history of traumatism unless a German sand soap which he used could have been the cause. The patient died.

Dr. William Green said he was much at sea about this case He was sent for one Sunday and found the patient up and about. Did not think much was the matter, left a prescription, and said he would return soon. On Tuesday he called to see his wife and was informed that he had gone to business. On Wednesday thought he was bilious and prescribed quinine. On the following Sunday he was sent for again, but his patient did not seem very sick. The dose of quinine was increased. On Wednesday the patient complained of feeling very tired and in the afternoon he showed some inco-ordination in the arm on attempting to shake hands. There was no fever, the pulse was 72, tongue clean and digestion good and only a feeling of general malaise. Dr. Chew was called in and by that time it was necessary to give chloroform to keep the patient quiet. He had opisthotonos and an aversion to liquid, but at no time did he have trismus to any extent and he could open his mouth, partially at all times except just before his death. There was no specific history.

Dr. F. T. Miles thought it was a wonderful case. The inco-ordination was out of the range of tetanus, and the want of trismus was remarkable. The difficulty of swallowing was sometimes caused by an occlusion of the glottis and did not always indicate an unwillingness to swallow.

Dr. J. J. Chisolm spoke of the profuse sweating which Dr. Green said came on at last.

Dr. S. C. Chew said there was no history of traumatism about the face as is usually the case. In reply to Dr. H. P. C. Wilson he said that the inco-ordination came on five hours before death. He referred to a case which he had seen with Dr. Miles. It was a case of acute bulbar paralysis developing with great rapidity. Antisyphilitic treatment cured it.

Dr. Christopher Johnston had used conine in half-drop doses.

Dr. H. P. C. Wilson had seen many cases of tetanus, but had never seen a case of idiopathic tetanus, and had had ample opportunities, in a large hospital dispensary and private practice, of observing many cases. He thought chloroform was the best treatment.

Dr. T. A. Ashby reported a complete recovery of his case of removal of the uterine appendages described at the last meeting.

Dr. J. J. Chisolm then read a paper entitled,

A VERY VALUABLE LESSON FOR THOSE WHO USE CHLOROFORM.

In the discussion which followed Dr. F. T. Miles spoke of the stimulating centres of the heart. He said the centre in the medulla was inhibitory and if stimulated the respiration would be inhibited. The cardiac centres were stimulated by the venous blood. Brown-Sequard recommended that the nose and mouth be stopped to stimulate the cardiac centres. In reversing patients in chloroform narcosis he thought that the effect of the large amount of blood below the thorax was stimulating. The liver contained one-fourth of the blood of the body and the hepatic veins could not collapse and thus the blood was carried directly into the cardiac cavities from auricles to ventricles.

Dr. S. C. Chew congratulated Dr. Chisolm on his boldness of action. He saw a case not long ago in which inversion was of no avail. Fleuron as well as Nelaton had made observations in this direction. When the heart ceased to act at once this method was of no use.

Dr. H P. C Wilson had used it many thousand times and had fortunately never had a fatal case. Had had patients very near death, but inversion with other remedies had been successful. Time was often lost in giving remedies. He had seen an unpleasant case in Liverpool in the service of Dr Alexander. The patient would have died but Dr. Wilson suggested inversion and restoration was complete. Chloroform was almost universally used in England.

Dr. Hiram Woods in speaking of the modes of death from chloroform, as described by Dr. Richardson, said that when the heart stopped suddenly all was at an end, and reversion then was without avail. It was important to know when to invert and it was also important to direct more attention to the countenance of the patient. In the case just reported Dr. Belt gave the chloroform and Dr. Randolph held the pulse, and yet both Dr. Belt and Dr. Woods observed a change in the face before anything was perceptible at the pulse. The capillaries might indicate a change more quickly to the sight than the radial pulse to the finger. The method of inversion was good in slow heart failure in which if let alone the patient would surely die. In this case the man had a strong regurgitant murmur. What would have been safer than chloroform?

